# Older assistant workers in intermediate care facilities, and their influence on the physical and mental burden of elderly care staff

**DOI:** 10.1186/s12913-021-07302-6

**Published:** 2021-11-30

**Authors:** Ryota Sakurai, Saya Watanabe, Hiroki Mori, Tomoya Sagara, Hiroshi Murayama, Shuichiro Watanabe, Kentaro Higashi, Yoshinori Fujiwara

**Affiliations:** 1 Research Team for Social Participation and Community Health, Tokyo Metropolitan Institute of Gerontology, 35-2 Sakae-cho, Itabashi-ku, Tokyo, 173-0015 Japan; 2 Graduate School of Gerontology, J. F. Oberlin University, 3758 Tokiwa-machi, Machida-shi, Tokyo, 194-0294 Japan; 3 Japan Association of Geriatric Health Services Facilities, 2-6-15, Shiba-koen, Minato-ku, Tokyo, 105-0011 Japan; 4 Mie Prefecture Association of Geriatric Health Services Facilities, Kawage-cho, Higashichisato, Tsu-shi, Mie 510-0303 Japan

**Keywords:** Elderly care home, Emotional exhaustion, Older adults, Work, Retirement

## Abstract

**Background:**

As there is a shortage of care staff in elderly care homes, seniors are expected to work as assistants to help the care staff. This study examined the influence of older assistant workers in intermediate elderly care facilities on care staff, specifically focusing on emotional exhaustion which is a sign of burnout. These facilities provide long-term nursing and supportive care to older residents.

**Methods:**

Data from a mail survey of intermediate elderly care facilities with older assistant workers were analyzed. Care staff were asked about the advantages and disadvantages of introducing older assistant workers in elderly care work, and their degree of emotional exhaustion. We also assessed work self-evaluations of older assistant workers, including the benefits of the work, and physical and mental burdens.

**Results:**

A significantly large number of care staff reported improvements in workload with the employment of older assistant workers. Intermediate elderly care facilities enrolling more older assistant workers showed lower mean emotional exhaustion among care staff, independent of possible covariates. While older assistant workers felt that their work contributed to helping both care users and staff, they also reported a mental burden.

**Conclusions:**

Our results suggest that older assistant workers can play a significant role in reducing the physical and mental burden of intermediate elderly care facility staff. Thus, employing older assistant workers can be an effective approach to addressing shortages of care staff in elderly care homes.

## Introduction

An ageing population and low birthrates are critical social issues in several developed countries. An important global issue due to a burgeoning aging population is the critical shortage of care professionals. In Japan, the country with the largest aging population, an estimated 2,500,000 elderly care workers will be needed in 2025 (the time that all baby boomers will become over 75 in Japan); the corresponding shortage of caregivers in elderly care homes is estimated to be approximately 340,000 workers [1]. This situation is similar to that in Western countries. For instance, 160,000 care staff will be needed to meet the demands of elderly care in England by 2032 [2]. By 2050, the number of older adults who need long-term care is projected to approach 15,000,000 in the US; a large number of care staff are required to support seniors [3].

Excessive workloads, including long working hours and high levels of stress, are reportedly the most important reasons for job dissatisfaction among care staff and nurses working in elderly care homes [4–6]. This suggests that early initiatives for reducing the physical and mental workload of elderly care staff are necessary to retain care staff, and maintain this workforce. This heavy workload results in job dissatisfaction, including reduced time for the elderly user (patient) and insufficient staffing levels [5]. Therefore, reducing workloads, including nonprofessional work (i.e., incidental tasks such as cleaning and waiting), may enable care staff to focus on their professional care work, thereby leading to fewer employees leaving the care work. One possible measure to maintaining the care workforce is effectively using an assistant worker who helps regular care staff and contributes to reducing their nonprofessional work.

Recently, the value and competence of older workers in the workforce of elderly care homes have received attention. If they can help elderly care staff by doing nonprofessional work, this can help reduce the physical and mental burden of the elderly care staff. This seems a reasonable measure for older workers because many of them are willing to work after they retire from their current jobs. Furthermore, working in later life can help them maintain their health [7–9]. Work in elderly care homes is regarded as very socially meaningful by older workers [10]. This perception may help improve or maintain their health because working for motivations other than financial reasons, such as finding meaning in life, is meaningful after retirement [11, 12]. Working in an elderly care home in old age may thus benefit both care staff and older workers; however, it remains unclear whether employing older people can actually reduce the physical and mental burden of care staff.

Poor physical work environments or badly designed and demanding work conditions leave people worn-out, resulting in earlier retirement. During this process, people generally experience greater emotional exhaustion, a state of feeling emotionally worn-out, and drained due to accumulated stress from their personal or work lives. This is especially true among clinical and care staffs [13]. Staff working in elderly care are exposed to a large number of factors that can lead to increased emotional exhaustion, which is a sign of burnout [14]. For example, they experience stress from heavy workloads, time pressure, dealing with people in need of long-term care, gaps between low salaries and high work demands, and staff shortages [5, 13, 15]. A lack of support and recognition among colleagues also contributes to greater emotional exhaustion [5, 16]. If older assistant workers can help reduce care staff workloads while developing mutual relationships, it may result in lower emotional exhaustion of care workers.

The present study thus aimed to evaluate the influence of older assistant workers on the physical and mental burden of elderly care staff to develop a deeper understanding of the potential contribution of working beyond retirement age in the care labor force. To determine whether older assistant workers contribute to reducing the physical and mental burden of care staff, which may decrease their emotional exhaustion, we conducted a mail survey focusing on intermediate elderly care facilities that have introduced older assistant workers in nursing homes [17]. Intermediate elderly care facilities (called Kaigo Rojin Hoken Shisetsu in Japanese) are long-term care facilities that provide nursing and supportive care, on a non-full-time and non-intensive nursing care basis, to older residents who are unable to care for themselves because of mental disability or declining health. We hypothesize that older assistant workers will receive favorable ratings from care staff in terms of reductions in workload, and thus, a larger workforce of older assistant workers will be associated with lower emotional exhaustion among care staff. This study also investigated the job descriptions, subjective benefits, and physical and mental burden of the work on older assistant workers. This study significantly contributes not only to addressing the shortage of care professionals but also to the development of policies and programs that facilitate social engagement among older adults.

## Material and methods

### Study design and subjects

Data were collected from a mail survey of nursing homes that have introduced an older assistant worker in 18 towns located in Mie Prefecture. This province has a working-age population ranging from 15 to 64 years old of 1,020,103 and an old-age population of those 65 years and above of 522,588 as of April 2019 (the ratio of older adults in the total population is 29.4%). This prefecture has implemented a model project from 2015 as a strategic policy to encourage the introduction of older assistant workers aged 60 and above engaged in nonprofessional care work, such as cleaning and keeping watch [18]. We defined those who were hired in this model project as older assistant workers.

From the 77 intermediate elderly care facilities in Mie Prefecture, our sample included 44 intermediate elderly care facilities that participated in the model project between 2015 and 2019, and had hired older assistant workers. Before the survey, the Association of Geriatric Health Services Facilities (i.e., the head office of the intermediate elderly care facility) asked each intermediate elderly care facility to participate in this survey via email. Questionnaires were sent to each intermediate elderly care facility, separately for the facility manager, care staff (full-time and part-time staff), and older assistant workers based on worker registrations. The facility manager was asked to distribute each questionnaire to each staff member.

### Questionnaire

#### Facility manager

The questionnaire for the facility manager included questions about the number of care staff and older assistant workers. The aim was to obtain the ratio of enrollment of older assistant workers in each intermediate elderly care facility.

#### Care staff

The survey questionnaire for care staff covered the following two topics: (1) perceptions of the advantages and disadvantages of introducing older assistance workers in intermediate elderly care facilities, and (2) the degree of emotional exhaustion. Question (1) applied to those who had experience working with older assistant workers, while question (2) applied to all care staff. Their sociodemographic variables were also assessed, including length of service. This is considered a particularly important covariate for care staff’s emotional exhaustion because 65.2% of care workers leave care work within three years in Japan, indicating that they are likely to show greater emotional exhaustion [5].

To understand the perceptions of the advantages of introducing older assistance workers, care staff answered six questions regarding improvements in workload, working environment, and mental health due to interactions with older assistant workers. Each question included six choices, ranging from “*Strongly agree*” to “*Strongly disagree*”. Participants were then classified as whether they felt the advantage (i.e., *Strongly agree* to *Somewhat agree*) or did not feel the advantage (i.e., *Somewhat disagree* to *Strongly disagree*) for each question. The perceptions of the disadvantages were assessed by two questions regarding concerns over accidents and decreased work efficiency similar to the questions regarding advantages.

The degree of emotional exhaustion was evaluated using the Emotional Exhaustion subscale of the Japanese version of the Burnout Questionnaire [19], which was based on modifying the Maslach Burnout Inventory [20]. The Emotional Exhaustion subscale consists of five questions (“Feel like at the end of the rope”; “Feel used up at the end of the workday”; “Feel fatigued when getting up in the morning”; “Feel emotionally drained from work”; and “Feel burned out from work”) scored on a scale ranging from 5 to 25; higher scores indicated greater emotional exhaustion. This subscale has been found to be reliable and has been previously validated for measuring burnout feelings [19]. Furthermore, the construct and factorial validity of the subscale assessing emotional exhaustion have been confirmed [21], particularly for medical workers [19, 22].

#### Older assistant workers

The survey questionnaire for older assistant workers covered the following topics: (1) current job description, (2) benefits of the work, and (3) physical and mental burdens of the assistant work as well as sociodemographic variables (e.g., work experience of care giving before starting the assistant work and subjective health).

The benefits of getting a job as a nursing assistant were assessed with six items scored on a four-point scale, ranging from “*Agree*” to “*Disagree*”, and classified as feeling the benefit (i.e., *Strongly agree* to *Somewhat agree*) or not feeling the benefit (i.e., *Somewhat disagree* to *Strongly disagree*). The degrees of physical and mental burdens of the assistant work were assessed on a five-point scale, ranging from “*A heavy burden*” to “*Not a burden*”. Participants were then classified as burdened (*Heavy burden* and *Somewhat burden*) or not burdened (*Neither*, *Less burden*, and *Not burden*).

### Data analysis

The characteristics of care staff and older assistant workers were summarized using frequencies and percentages. Differences in dichotomized questions were analyzed using one-sample chi-square tests. For emotional exhaustion among care staff, we first calculated the mean ratio of enrollment of older assistant workers in each intermediate elderly care facility as follows: (number of older assistant workers/(number of care staff, including older assistant workers))*100. Then, partial rank correlation analysis was applied because of the non-parametric distribution. This analysis was adjusted for the number of care staff, mean female ratio, and mean ratio of nursing care staff who work in a current intermediate elderly care facility for 3 years or more. All statistical analyses were performed using IBM SPSS Statistics (version 21.0; SPSS Inc., Chicago, IL), with the level of significance set at *p* < 0.05.

## Results

Questionnaires were mailed to 44 intermediate elderly care facilities, and 33 facilities responded to the survey (response rate = 75.0%). The total number of completed questionnaires returned by facility managers, care staff, and older assistant workers were 30, 844, and 62, respectively. The characteristics of care staff and older assistant workers are shown in Table 1. More than 70% of the care staff were female, worked full-time, and had work experience at their current intermediate elderly care facility for 3 years or more. Most older assistant workers did not have care-related work prior to their current work and had good subjective health.

**Table 1:** Characteristics of nursing care staff and older assistant workers

	Nursing care staff	Older assistants
	*n* = 844	*n* = 62
Female, *n* (%)	597 (70.7)	53 (85.5)
Age, *n* (%)		
≤ 29 yrs., *n* (%)	177 (21)	
30–39 yrs., *n* (%)	206 (24.4)	
40–49 yrs., *n* (%)	202 (23.9)	
50–59 yrs., *n* (%)	165 (19.5)	
≥ 60 yrs., *n (%)*	94 (11.1)	
60–64 yrs., *n* (%)		9 (14.5)
65–69 yrs., *n* (%)		22 (35.5)
70–74 yrs., *n* (%)		25 (40.3)
75–79 yrs., *n* (%)		6 (9.7)
3 yrs. or more of work experience^a^, *n* (%)	597 (70.8)	
Full-time staff, *n* (%)	639 (76.1)	
Working with older assistants, *n* (%)	275 (32.6)	
Work experience of care^b^, *n* (%)		5 (8.1)
Good subjective health, *n* (%)		54 (87.1)

^a^ Three years or more of working in a current intermediate elderly care facility

^b^ Work experience of care before working as an assistant

### Subjective evaluation by care staff on working with older assistant workers

**Fig. 1:**
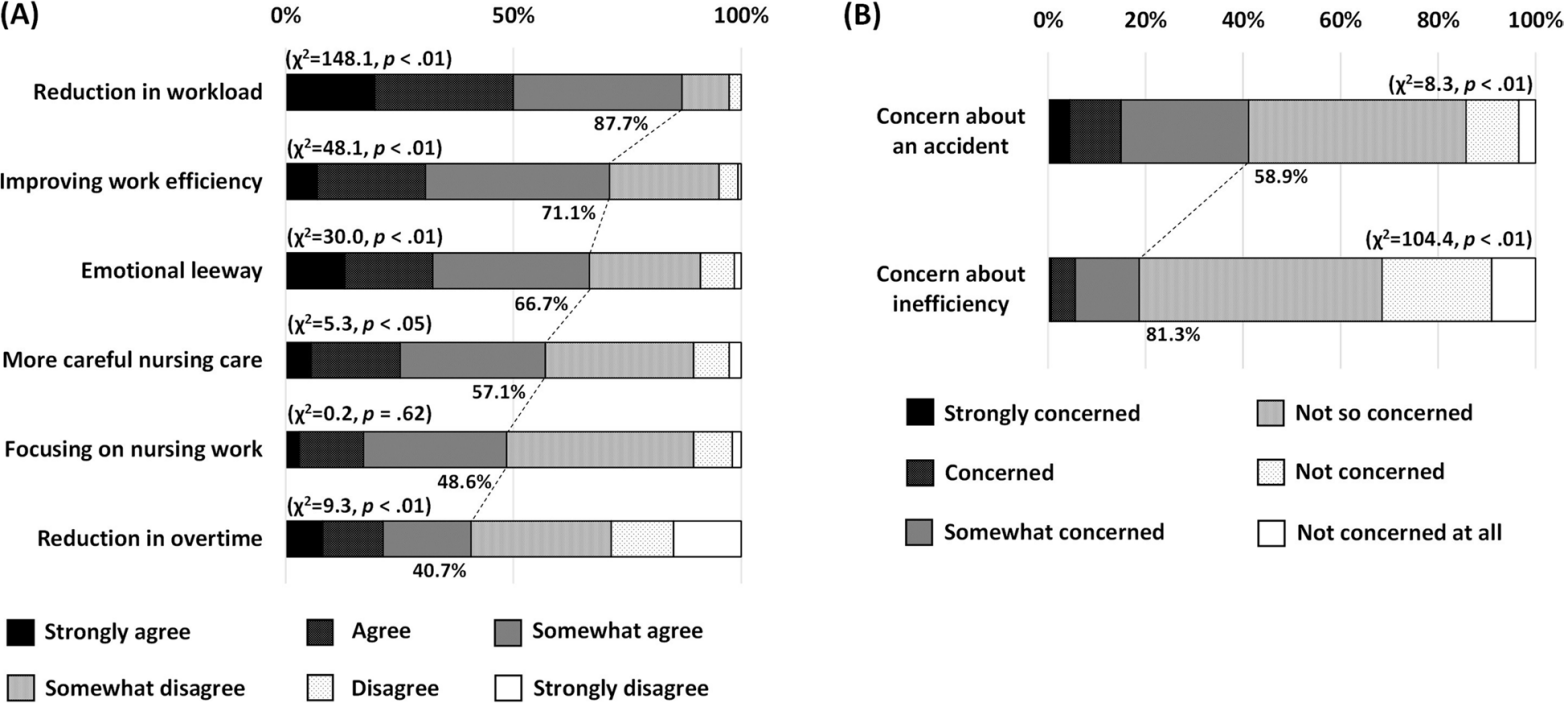
Subjective results for the nursing staff regarding (**A**) advantages and (**B**) disadvantages of introducing older assistant workers in intermediate elderly care facilities. **A** Answers were classified as feeling advantage (*Strongly agree* to *Somewhat agree*) or not feeling advantage (*Somewhat disagree* to *Strongly disagree*) for each question, and then one-sample chi-square tests were performed. **B** Answers were classified as concerned (*Strongly concerned* to *Somwhat concerned*) or not concerned (*Not so concerned* to *Not concerned at all*) for both questions, and then one-sample chi-square tests were performed

A total of 275 care staff members had experience working with older assistant workers, and answered the question regarding perceptions about the advantages and disadvantages of introducing older assistance workers into intermediate elderly care facilities. As shown in Fig. 1A, many participants responded that there are some advantages of working with older assistant workers (*p* < .01 or .05), except for focusing on care work and reduction in working overtime. More than 70% of the care staff reported that older assistant workers contributed to a reduced workload and improved work efficiency. Furthermore, staff were not concerned about older assistant workers experiencing accident or being inefficient (Fig. 1B; *p* < .01).

**Fig. 2:**
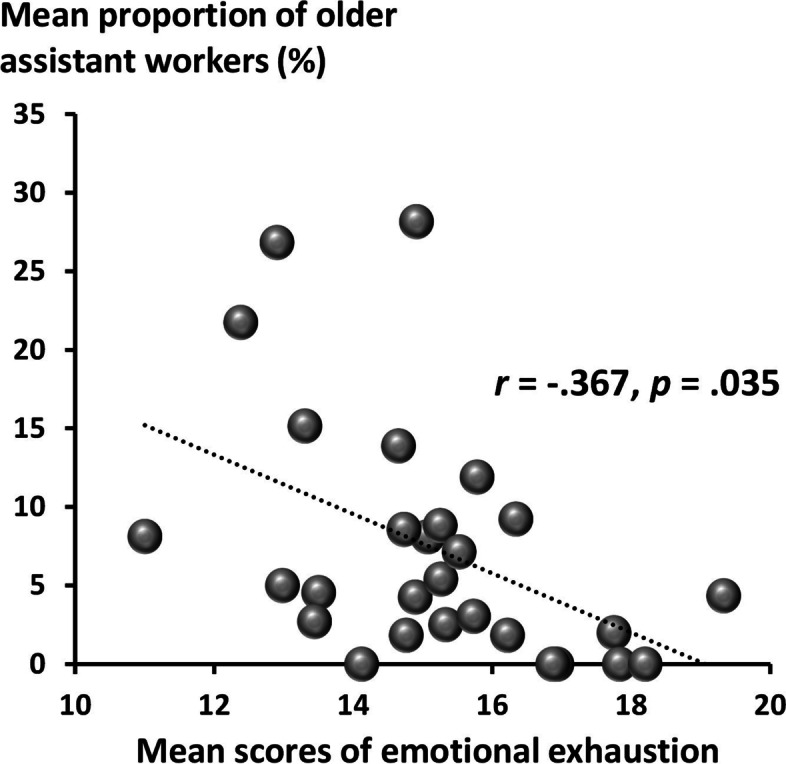
Scatter diagram of the mean proportion of older assistant workers and mean scores of the care workers’ emotional exhaustion. Each plot indicates each facility

Figure 2 shows a scatter diagram of the ratio of enrollment of older assistant workers and the mean emotional exhaustion among care staff in each intermediate elderly care facility. Among the facilities that responded to the questionnaire (*n* = 33), three facility managers returned the questionnaires in blank and two responses were incomplete on the number of care staff. Thus, partial correlation analysis assessing the association between the ratio of enrollment and mean emotional exhaustion was performed on responses from 28 facilities (777 nursing care staff members). The results of the partial correlation analysis, adjusting for potential covariates (the number of care staff, mean female ratio, and mean ratio of nursing care staff who work in a current intermediate elderly care facility for 3 years or more), showed a significant negative correlation. This indicates that intermediate elderly care facilities with more older assistant workers tended to show lower mean emotional exhaustion among care staff (*r* = −.367, *p* = .035).

### Subjective evaluation of older assistant workers regarding their work

**Fig. 3:**
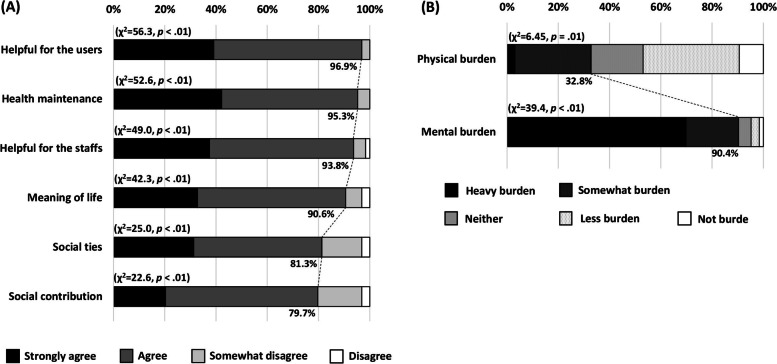
Subjective results for older assistant workers regarding (**A**) benefits, and (**B**) physical and mental burden of the assistant work. **A** Answers were classified as a feeling benefit (*Strongly agree* to *Somewhat agree*) or not feeling benefit (*Somewhat disagree* to *Strongly disagree*) for each question, and then one-sample chi-square tests were performed. **B** Answers were classified as burdened (*Heavy burden* and *Somewhat burden*) or not burdened (*Neither*, *Less burden*, and *Not burden*) for both questions, and then one-sample chi-square tests were performed

The top five tasks older assistant workers performed were serving meals and cleaning the Table (54.0%), cleaning (50.8%), making beds (50.8%), keeping watch (46.0%), and active listening (41.3%). Many older assistant workers agreed on the benefits of the work (Fig. 3A; *p* < .01); more than 90% felt that their work contributed to helping both users and staff, maintaining their health, and finding meaning in their lives. Figure 3B shows the subjective physical and mental burdens of older assistant workers. Many of them answered that they felt a mental burden from the work (90.4%; *p* < .01), but not physical burden (32.8%; *p* = .01).

## Discussion

This study showed that older assistant workers received favorable ratings from care staff in terms of reductions in workload and improved work efficiency. Moreover, intermediate elderly care facilities with a higher ratio of older assistant workers tended to show lower mean emotional exhaustion among care staff. Our results support our hypotheses and suggest the effectiveness of older assistant workers in intermediate elderly care facilities in reducing the workload of care staff and opens up new possibilities in compensating for labor shortages in elderly care homes.

We found that a larger workforce of older assistant workers was independently associated with lower emotional exhaustion among care staff. One possible explanation for this favorable association may be a reduction in the overall workload, leading to improved work efficiency. This is evident in the results of perceptions about the advantages and disadvantages of introducing older assistance workers (Fig. 1A). Furthermore, care staff might receive emotional support from older assistant workers. Several forms of support provided by older people can reduce depressive symptoms and increase self-esteem in other generations [23, 24]. These effects of intergenerational relationships may mediate this association. Still, further studies are needed to examine the underlying mechanisms of this association.

Some suggest that there is a risk of accidents when working in old age [25]. This is prominent in healthcare settings because the recipients of services are frail people, including the disabled elderly. However, more than half of the care staff who had experience working with older assistant workers were not concerned about accidents in their work (58.9%). This may be due to the employment procedure for older assistant workers at the study site. In the model project conducted in Mie prefecture, to encourage the introduction of older assistant workers, healthy seniors were recruited and received a full explanation of their work in advance. More importantly, work in the intermediate elderly care facility was isolated: professional care work was undertaken by nursing care staff and nonprofessional care work was undertaken by older assistant workers. Therefore, older assistant workers engaged in their work safely, and consequently, nursing care staff were not anxious about accidents in the assistants’ work.

We found that older assistant workers find value in working in intermediate elderly care facilities in terms of contributions to both care users and staff. However, older workers felt mental burden, but not physical burden, from their work. Considering the type of work they perform, this is a natural result because working in an elderly care home generally requires delicate care, resulting in stress. Although moderate stress exposure facilitates resilience, considering the nature of working after retirement age, such as working for not only financial reasons but also for greater meaning of life [11, 12], measures are needed to reduce the mental burden of older assistant workers. This may eventually result in a larger enrolment of older assistant workers in intermediate elderly care facilities.

This study offers many policy and practical insights for expanding the elderly care labor force using older assistant workers. However, there are some limitations that must be considered when interpreting these results. First, our sample came from specific intermediate elderly care facilities that participated in the model project and had hired older assistant workers. This may limit the generalizability of our results due to poor heterogeneity. Second, the cross-sectional design precludes us from exploring causal relationships between the associations we found and the long-term effects of older assistant workers. Further longitudinal research is needed to examine the long-term effects of introducing older assistant workers into intermediate elderly care facilities, including the effect on elderly users (patients). Future studies can also examine the applicability of onboarding older assistant workers to other workplaces with labor shortage issues.

## Conclusion

The present study demonstrated that older assistant workers can play a role in reducing the physical and mental burden of care staff without increased concerns about accidents.

Our results underscore the possibility that the employment of older workers can be an effective measure against the shortage of care staff in elderly care homes. There may be a need to develop an older workforce in a way that helps compensate for the shortage of frontline workers in elderly care homes.

## Data Availability

The dataset used is owned by the Ministry of Health, Labour and Welfare, and sharing the data is restricted. However, data may be available from the authors upon reasonable request and with permission from the Ministry. Please find the contact information where you can direct data inquiries here: kenkyurinri@tmghig.jp (Tokyo Metropolitan Institute of Gerontology Ethics Board). The questionnaires used in this study are also available from the corresponding author upon request.
